# Effects of Treatment Preference on Adherence, Attrition, and Process Measures Among Older Adult Worriers

**DOI:** 10.1093/geroni/igaa057.1192

**Published:** 2020-12-16

**Authors:** Gretchen Brenes, Heidi Munger Clary, Michael Miller, Jasmin Divers, Andrea Anderson, Gena Hargis, Suzanne Danhauer

**Affiliations:** 1 Wake Forest School of Medicine, Winston-Salem, North Carolina, United States; 2 NYU Winthrop Hospital, New York, United States

## Abstract

Patient preference may be related to treatment outcomes through decreased rates of attrition and higher rates of adherence and patient satisfaction. We present findings from a 2-stage randomized preference trial of cognitive-behavioral therapy (CBT) and yoga for the treatment of late-life worry. We examine rates of preference for CBT and yoga, as well as the stability of these preferences over time. We also examine the impact of preference on adherence, attrition, and process measures (satisfaction, treatment expectancies, and working alliance). Five hundred participants were randomized to either the randomized controlled trial (RCT; N=250) or the preference trial (participants chose the treatment; N=250). All participants received 10 weeks of an intervention. Among those in the preference trial, 48% chose CBT and 52% chose yoga (p>.05). Strength of preference was similar between the groups; 73.3% and 76.2% reported a strong preference for CBT and yoga, respectively (p>.05). Fourteen percent of those who preferred CBT at baseline preferred yoga upon completion of the intervention, while 12.2% of those who preferred yoga at baseline preferred CBT upon completion of the intervention (p>.05). There were no significant differences between participants in the RCT and preference trial on intervention adherence, attrition, satisfaction, or working alliance (p’s>.05). Treatment expectancies were higher for the preferred intervention (p’s<.0001). Results suggest that older adults prefer CBT and yoga at similar rates, and these preferences are stable. Receiving a preferred treatment had no effect on adherence, attrition, satisfaction, or working alliance.

